# Roles of Metabolites Unveiled by Metabolomics in *Brassica rapa*, *B. napus* and *B. juncea*

**DOI:** 10.3390/metabo16060417

**Published:** 2026-06-15

**Authors:** Yunong Xia, Silin Su, Xianyu Tang, Lei Qin, Junxing Lu, Shitou Xia

**Affiliations:** 1 Hunan Provincial Key Laboratory of Phytohormones and Growth Development, College of Bioscience and Biotechnology, Hunan Agricultural University, Changsha 410128, China; yun0623@stu.hunau.edu.cn (Y.X.); susilin@stu.hunau.edu.cn (S.S.); chuxuantingnasha@stu.hunau.edu.cn (X.T.); 2 Yuelushan Laboratory, Changsha 410125, China; qinlei@hunaas.cn; 3 Crop Research Institute, Hunan Academy of Agricultural Sciences, Changsha 410125, China; 4 Chongqing Key Laboratory of Plant Environmental Adaptations, College of Life Science, Chongqing Normal University, Chongqing 401331, China; junxlu@163.com

**Keywords:** metabolite, rapeseeds, metabolomics, growth and development, abiotic/biotic stress, resistance

## Abstract

Rapeseed is a major source of vegetable oil and contains a wide variety of metabolites. Recent advances, particularly the integration of metabolomics with other omics approaches, have enabled not only comprehensive but also detailed analyses of key metabolites that respond to specific conditions. To date, these recent advances in the metabolomics of *Brassica* crops have not yet been fully clarified. In this review, we seek to summarize the recent progresses in metabolomics studies of *Brassica rapa*, *B. napus* and *B. juncea*, introduce the key metabolites spanning nucleic acids, amino acids, fatty acids, lipids, organic acids, alkaloids, phenylpropanoids, terpenoids, flavonoids and glucosinolates uncovered by this approach, focusing on those associated with growth and development, and abiotic/biotic stresses, including macronutrient availability, temperature, water stress, salt stress, aluminum and cadmium toxicity, and infection of *Sclerotinia sclerotiorum*, *Leptosphaeria maculans*, and *Plasmodiophora brassicae*. Future perspectives and current challenges in metabolomics integrating with other omics are also discussed, along with its potential for breeding applications, especially in new marker discovery, trait prediction, and even metabolic selection, aimed at developing new rapeseed varieties with stable, high-yielding, and quality traits.

## 1. Introduction

Rapeseed is the world’s second-largest oil crop and plays a significant role globally in agricultural economies, which is composed of *Brassica rapa* (AA), *B. napus* (AACC), *B. juncea* (AABB), and *B. carinata* (BBCC) [1]. Of which, the allopolyploid *B. napus* (*n* = 19) and *B. juncea* (*n* = 18) are widely grown in Europe, Canada, South Asia, China, and Australia [2,3]. As the main source of vegetable oil, rapeseed contributes approximately 13–16% of the total global edible vegetable oil production [4].

Metabolites are the products comprising precursors, intermediates, and final products of cellular regulatory processes, reflecting the physiological state of plants at the metabolic level. As the direct cause of phenotype changes, metabolites reveal more comprehensively the function of genes, linking the upstream gene function and the downstream phenotype changes [5,6]. There are various types of plant metabolites, which can be mainly divided into two categories: primary metabolites and secondary metabolites, contributing the most metabolite diversity with 200,000–1,000,000 types of metabolites [7,8,9,10,11]. Plant metabolites are not only essential for growth and development but also serve as key functions in environmental defense and adaptation [12,13].

A biological system synthesizes a set of metabolites that constitutes its “metabolome”. Based on metabolome, Fiehn [5] proposed “metabolomics” in 2001 and defined it as “the comprehensive qualitative and quantitative analysis of all metabolites in a biological system”. According to the purpose and application of the research, metabolomics can generally be categorized into targeted and untargeted approaches. The former aims to quantitatively verify the expression differences in specific metabolites, mainly used to further analyze the biological functions of known biochemical pathways [6,14], while the latter aims to study metabolites that have significant statistical differences between experimental and control groups, and more from a qualitative and semi-quantitative perspective to discover differences in metabolomics. Therefore, non-targeted metabolomics can be used to discover various new biomarkers and even the entire metabolic pathway [15,16]. At present, most of the metabolomics research is focused on the combination of targeted and non-targeted approaches, from “discovery” to “validation”, in order to better utilize the role of metabolomics. Since its introduction in the 1990s, metabolomics has found broad applications in plants, enabling the detection of new metabolites, genes, and metabolic pathways [17]. Increased evidence shows that metabolomics offers a powerful means of better understanding how plant metabolites are synthesized and how they function [18,19,20,21].

*Brassica* crops are rich in types of metabolites; studying the changes and regulation of metabolites as a whole can lay the foundation for analyzing plant growth and development, as well as their interactions with environmental factors. The powerful multi-omics techniques and analytical tools have enabled the efficient identification of numerous genetic loci and candidate genes linked to important traits [22]. Although several reviews about omics studies in abiotic stress regulation [23,24], host–pathogen interactions in *B. napus* [25,26], or major oilseed crops [27] were published, very recent advances in metabolomics of the *Brassica* crops, comprising *Brassica rapa*, *B. napus*, and *B. juncea*, have not yet been fully clarified to date. With the rapid widespread application of metabolomics, more and more important agronomic, physiologic, and nutritional traits, such as embryo development, seed oil accumulation, seed coat color, abiotic/biotic stress resistance, and related metabolites, have been reported in rapeseeds. Here, we seek to summarize the main progress in metabolomics research of rapeseed in recent years, focusing on the metabolites unveiled by metabolomics and their functions in growth and development and defense against both abiotic and biotic stresses.

## 2. Analyses of Metabolites by Metabolomics

Qualitative and quantitative analysis of primary and secondary metabolites in samples can reflect the cellular state of rapeseeds under specific conditions, which helps to gain a deeper understanding of the cellular-level processes that regulate the biochemical phenotype of organisms. Benefiting from technological advances enabled by these strategies, thousands of metabolites have been identified, encompassing sugars, nucleic acids, fatty acids, lipids, amino acids, organic acids, alkaloids, phenolic acids, terpenoids, phenylpropanoids, flavonoids, and glucosinolates. For instance, Li et al. [28] uncovered 2173 metabolites in the mature seeds of 388 *B. napus* accessions, including nucleic acid derivatives (**38**), amino acid derivatives (**80**), lipids (**60**), phenylethanoids (**29**), terpenoids (**42**), flavonoids (**92**), and other metabolites (**99**). Using six varieties from each of the three species *B. rapa*, *B. napus*, and *B. juncea* collected from different regions of China, Zhang et al. [29] identified a range of compounds: flavonoids (**36**), coumarins (**23**), phenolic acids (**12**), lignans (**10**), stilbenes (**4**), diarylheptanes (**4**), and tannin (**1**). Among these three rapeseed genotypes, *B. napus* consistently showed the highest levels of total phenolics and flavonoids, as well as the highest antioxidant capacity.

Upon profiling the metabolomes of yellow and black seeds in diverse rapeseed accessions, a total of 248 features, comprising glucosinolates (**24**), phenolic acids (**31**), flavonoids (**54**), lipid compounds (**65**), and other polar compounds (**74**), were identified [30]. Similarly, Li et al. [31] found a total of 531 metabolites in Chinese cabbage planted in the Jiaozhou and Jinan areas. Among these, 529 were present in samples from both growing regions, and 108 were associated with traditional Chinese medicine. Rich in various nutrients, Chinese cabbage contains saccharides, lipids, amino acids and their derivatives, organic acids, phenolic acids, nucleotides and their derivatives, glucosinolates, flavonoids, and vitamins. Comparative investigation revealed regional differences in metabolic profiles. Of the 89 differentially altered metabolites (DAMs) characterized, 78 were more abundant in the Jinan area, while 11 were higher in the Jiaozhou area [31]. S-(methyl) glutathione and nicotinic acid adenine dinucleotide were two metabolites uniquely detected in the Jiaozhou area [31].

Furthermore, when a core set of 168 accessions of *B. rapa*, representing diverse morphotypes types and geographical origins, was investigated to identify hereditary associations between genetic markers and key metabolites including carotenoids, tocopherols, and chlorophylls, Pino et al. [32] obtained a set of significant markers, linked to the metabolites, which could be potentially used to select genotypes that exhibit higher levels of these compounds. Three years later, Pino et al. [33] profiled the secondary metabolites of 6-week-old leaves from 92 *B. rapa* doubled haploid genotypes, using both targeted and LC-MS-based untargeted metabolomics approaches. Colocalization of both metabolite QTLs (mQTLs) and expression QTLs (eQTLs) pinpointed candidate regulatory genes governing the biosynthesis of tocopherols, carotenoids, and glucosinolates. Thereafter, the authors identified two hotspots on linkage groups A03 and A09 where eQTLs co-localized with mQTLs in the glucosinolate biosynthesis pathway [33].

Many *Brassicaceae* species produce large quantities of soluble hydroxycinnamate conjugates, mainly sinapate esters, via the phenylpropanoid pathway. In *B. napus*, *REDUCED EPIDERMAL FLUORESCENCE1* (*REF1*) encodes a coniferaldehyde/sinapaldehyde dehydrogenase involved in converting the corresponding aldehydes into ferulate and sinapate, thus functioning as a potential branch-point enzyme that bridges lignin and hydroxycinnamate biosynthesis [34]. Suppression of *BnaREF1* in seeds led to a novel chemotype revealed by nontargeted metabolite profiling, including reduced sinapates, the emergence of conjugated mono- and di-trilignols, modifications in kaempferol glycoside patterns, and minor caffeate and ferulate conjugates, with minor sinapate conjugates being more affected than the major component sinapine. Mapping these metabolic changes onto the phenylpropanoid network further indicated that a partial redirection of metabolic sequences was a major consequence of *BnaREF1* suppression [34].

Recently, Yang et al. [35] established the BnIR database (http://yanglab.hzau.edu.cn/BnIR, accessed on 13 May 2026) for rapeseed, which houses six omics layers—genomics, transcriptomics, phenomics, epigenetics, variomics, and metabolomics, together with numerous “variation–gene expression–phenotype” associations using various statistical approaches. Additionally, the database integrates a suite of multi-omics search and analysis tools to enable easy browsing and application of these datasets. To date, BnIR represents the most comprehensive multiple omics database for rapeseed. Two case studies have validated its effectiveness in identifying candidate genes linked to specific traits and elucidating their potential regulatory mechanisms [35,36]. Another comprehensive rapeseed multi-omics database, BnaOmics, under the background of pan genomics, has also been established by the Oil Crop Research Institute of the Chinese Academy of Agricultural Sciences [37]. These databases provide important data support and retrieval analysis platforms for rapeseed genetic breeding.

## 3. Metabolites Related to Growth and Development

### 3.1. Embryo Development

The embryo is the core component of rapeseeds, accounting for over 80% of seed weight. However, due to the constrained development, the embryo must modify its size and shape to fit the confined space within the seed [38]. Borisjuk et al. [38] employed noninvasive NMR-based imaging to study developing *B. napus* seeds and revealed that gradients in lipid concentration become established after embryo bending. These gradients were found to correlate with the local ETR (electron transport rate) and storage product accumulation.

According to predictions of spatially resolved metabolic models, the outer cotyledon and hypocotyl/radicle produce most of their plastidic reductant and photosynthetic ATP, whereas the inner cotyledon, enclosed by the outer one, relies on heterotrophic growth [38]. Under field-like high light, a significant Rubisco bypass contribution to seed storage metabolism is predicted solely for the outer cotyledon and hypocotyl/radicle. Furthermore, comparisons between in planta and in vitro embryos reveal that in vivo metabolic fluxes are subject to local regulation and are intimately linked to seed architecture, promoting efficient light and space utilization by the embryo [38].

To investigate dynamic changes in protein abundance during seed development, Lorenz et al. [39] extracted the total soluble protein fractions from endosperm and embryos at specified time points. They found that proteins associated with the conversion and recycling of sugars, amino acid biosynthesis, ascorbate metabolism, and redox balance are particularly important for seed development in *B. napus*. In another study, Iwase et al. [40] reported that WOUND INDUCED DEDIFFERENTIATION1 (*At*WIND1) could enhance the transcripts of *B. napus* homologous genes of *ESR1/DRN* (*ENHANCER OF SHOOT REGENERATION1/DORNRÖSCHEN*), a direct transcriptional target of WIND1 in *Arabidopsis thaliana*. Furthermore, temporal profiling of phytohormones revealed a perturbed equilibrium between endogenous auxin and cytokinin in *B. napus* explants following *At*WIND1 activation. Additionally, dynamic metabolomic reprogramming was uncovered in these explants, including the buildup of compounds like Pro, putrescine, and GABA (gamma-aminobutyric acid), which have traditionally been added to tissue culture media to promote plant cell reprogramming [40].

### 3.2. Oil Accumulation

It appeared in general that many species of phosphatidylcholine (PC) and triacylglycerol (TAG) showed heterogeneous distribution within the entire embryo, as tissue-specific transcriptomics uncovered key genes for plastidial fatty acid synthesis, ER-based TAG assembly, and lipid droplet packaging, potentially underlying the high/low-oil trait and heterogeneous lipid patterns [41]. Using a widely targeted metabolomics approach on a natural population of 388 *B. napus* inbred lines, Li et al. [28] quantified 131 marker metabolites that were identified as correlated with seed oil concentration. Those metabolites were subsequently selected for further metabolite genome-wide association studies (mGWAS) and metabolite transcriptome-wide association studies (mTWAS). By integrating weighted correlation network analysis, the authors constructed a metabolite triple-relationship network with 4384 nodes and 21,000 edges, mQTLs, genes, and co-expression modules. The functions of three candidate genes predicted by this multi-omics analysis—*BnaA03.TT4*, *BnaC02.TT4*, and *BnaC05.UK*—were experimentally validated, demonstrating that they significantly impact seed oil concentration by regulating *B. napus* flavonoid metabolism and demonstrating the value of integrating marker metabolites with multiple omics analysis for dissecting the genetic basis of crop agronomic traits [28].

To explore the metabolic changes during oil accumulation, Tan et al. [42] extracted metabolites from both seeds and silique walls and then characterized them via GC-MS (gas chromatography–mass spectrometry). Across four developmental stages, 443 metabolites were identified in total. Of these, dozens exhibited differential expression throughout seed maturation, including 20 known seed development-related metabolites. Furthermore, Tan et al. [42] examined metabolic alterations in silique walls and seeds across three treatments: Ld (leaf detachment), Pe (phloem peeling), and Sd (selective silique darkening). Oil content was found to be dependent on metabolic influx from the silique wall, but not leaf photosynthesis and phloem transport during oil accumulation. Furthermore, Pe- and Sd-treated seeds unexpectedly accumulated organic acids and sugar derivatives. In line with these findings, Pe- and Sd-treated seeds showed significant changes in the expression of genes related to sugar storage, fatty acid metabolism, and oil storage [42].

Moreover, a trade-off was found to exist between protein and oil storage in *B. napus*. When key napin- and cruciferin-encoding genes were downregulated, Rolletschek et al. [43] found that soluble sugars, mainly sucrose, ranked as the most abundant metabolite category, with amino acids (notably Asp, Arg, Glu, and Gln) and organic acids (particularly malate and citrate) being the next most abundant. Of note, the amounts of polyamines and amino acids in transgenic embryos were approximately double those measured in wild-type segregant (WTS) embryos. Statistically significant increases were observed for Gln (163%), His (136%), Arg (118%), Val (38%), and Trp (34%), while strong but non-significant increases were noted for Asp (55%), citrulline (75%), Orn (184%), and Asn (192%). The level of oxaloacetate, which is generated by the PEPC reaction, was significantly elevated (24%) in transgenic embryos relative to their WTS counterparts. Storage protein suppression induced oleosin-enriched membrane stacks (sixfold increase) and a novel ER morphology, which helped maintain embryonic carbon/nitrogen homeostasis [43].

In addition, when leveraging previously published metabolome data from a population of 388 *B. napus* accessions, Li et al. [44] identified 137 marker metabolites significantly correlated with the thousand-seed weight (TSW), and 21 marker metabolites significantly correlated with both TSW and seed oil content. A comprehensive multi-omics analysis revealed 734 mQTLs, 5225 eQTLs, and 9077 transcriptome-wide significantly associated genes, highlighting the role of the transcription factor *Bna*A03.TGA6 (TGACG motif-binding factor 6) in influencing TSW. Furthermore, *tga6* mutants exhibited changed metabolite levels compared to wild-type plants and produced larger seeds, as demonstrated by functional validation experiments, unraveling the genetic basis of TSW in *B. napus* [44].

### 3.3. Seed Coat Color and Flavonoid Synthesis

To explore the mechanisms underlying seed coat color, Shen et al. [45] performed transcriptomic and metabolic analyses of dark and yellow seeds in *B. juncea*. The analysis revealed 236 compounds, comprising 17 glucosinolates, 31 phenolic acids, 38 lipids, 47 flavonoids, 69 additional hydroxycinnamic acid compounds, and 34 previously unknown compounds. Among those, 36 compounds, particularly epicatechin and its derivatives, exhibited significantly divergent accumulation profiles over the course of seed development between dark- and yellow-seeded *B. juncea* genotypes. Moreover, *BjuTT8*, *BjuTT19*, *BjuANS*, *BjuDFR*, and *BjuBAN* transcript levels closely tracked epicatechin and derivative changes across seed development, implicating this pathway in *B. juncea* seed color determination. In addition, sequence variations in *TT8A* were postulated to be linked to seed color stability in *B. napus*, *B. rapa*, and *B. juncea*, potentially having experienced functional divergence during the course of polyploidization within the *Brassica* genus [45].

Likewise, in an integrated metabolome, transcriptome, and genome-wide association study (GWAS) of different *B. rapa* varieties, Zhao et al. [46] identified 2499 genes exhibited differential expression, and 116 metabolites displayed differential accumulation, when comparing yellow and black seeds. These molecular features were strongly linked to the flavonoid biosynthetic pathway. Using weighted gene co-expression network analysis (WGCNA), the authors detected 330 core network genes involved in seed color determination, together with the most differentially expressed genes associated with flavonoid biosynthesis. mGWAS analysis, based on the accumulation levels of 42 distinct flavonoids in the developing seeds of 159 *B. rapa* accessions, identified 1626 QTNs (quantitative trait nucleotides), including a major locus located on the A09 chromosome. By integrating QTN identification, transcriptomic, and functional assays, researchers identified a total of 241 candidate genes showing association with various flavonoids. Using the set of identified metabolites and gene candidates, a reconstruction of the flavonoid biosynthesis pathway was performed in *B. rapa* [46].

When using UHPLC-MS/MS to investigate how salicylic acid (SA) regulates seed color of rapeseed, Xia et al. [47] demonstrated for the first time that increased levels of SA enhance anthocyanin accumulation within the seed coats of *B. napus*, thereby promoting black seed formation. Conversely, diminished SA levels facilitate the expression of the yellow-seeded trait. Furthermore, treatment with exogenous SA (1 mmol/L) led to a significant increase in pigmentation within seed coats of the yellow-seeded variety “Huang’ aizao” (HAZ), resulting in a higher proportion of dark brown seeds. For simplicity and convenience, typical metabolite types identified in rapeseeds through metabolomics up to now are summarized in Table 1.

Flavonoid biosynthesis genes typically fall into two categories: structural and regulatory [58]. The flavonoid biosynthetic-related genes at early steps, also known as early biosynthetic genes (EBGs), encode key enzymes including CHI (chalcone isomerase), CHS (chalcone synthase), F3H (flavanone 3-hydroxylase), F3′H (flavonoid 3′-hydroxylase), and F3′5′H (flavonoid 3′,5′-hydroxylase), which are involved in the biosynthesis of common precursor molecules [59,60,61,62]. In contrast, the late biosynthetic genes (LBGs) encode enzymes such as FLS (flavonol synthase), DFR (dihydroflavonol-4-reductase), LAR (leucoanthocyanidin reductase), ANR/BAN (anthocyanidin reductase), LDOX (leucoanthocyanidin dioxygenase, also known as anthocyanidin synthase, ANS), and UFGT (UDP-glucose:flavonoid 3-O-glucosyltransferase). These LBG-encoded enzymes catalyze the biosynthesis of anthocyanins, additional end products of the flavonoid pathway (namely flavonols and flavones), and phenolic acids [60,61,62,63].

The regulatory genes mainly consist of a set of transcription factor-encoding genes, including *TT1*, *TTG1*, *TT2*, *TT8*, and *TT16*, which are implicated in controlling the production of anthocyanins and proanthocyanidins (PAs) [58,64]. For instance, TT2 (an R2R3-MYB protein), TT8 (a bHLH protein), and TTG1 (a WD40 protein) [65] assemble into the ternary MBW (MYB–bHLH–WDR) complex. This complex critically regulates both the levels and spatial/temporal patterns of structural gene expression, thereby serving a crucial role in the flavonoid biosynthetic pathway [20,65,66,67].

Integrating transcriptome and WGCNA with anthocyanin metabolism data, Cui et al. [68] proposed that the upregulation of *MYB75* enhances *F3H* expression, thereby playing a key role in pigmented flower development in rapeseeds. Using multi-omics datasets, Zhang et al. [22] experimentally validated that *Bna*C07.CCR-LIKE (CCRL) and *Bna*TT8 positively regulate seed coat and lignin content. They also predicted key genes that govern the allocation of carbon resources between the biosynthesis of phenylpropane-derived compounds and oil during the course of seed development in *B. napus*. In another separate study, Knoch et al. [69] performed phenotypic data analysis on a diverse rapeseed population comprising 477 spring-type lines and reported 61,298 robust QTLs (quantitative trait loci), including mQTLs (187), eQTLs (56,814), and phenotypic QTLs (4297). Many of these QTLs were clustered in pronounced hotspots.

## 4. Metabolites Related to Abiotic and Biotic Resistance

Seeds of *Brassicaceae* species are capable of producing a diverse repertoire of specialized metabolites that are both beneficial and antinutritional, influencing seed quality and providing resistance to environmental stresses. Some primary metabolites, such as the nonenzymatic antioxidants ascorbic acid and carotenoids, play a protective role against the production of reactive oxygen species (ROS) in plants [70]. In contrast, a range of secondary metabolites, comprising flavonoids, terpenoids, phenolics, lignin, glucosinolates, and tannins, are recognized as markers for biotic and abiotic stress tolerance [70,71]. Rapeseed exhibits accumulation of flavonoids (such as anthocyanins, flavanols, flavanones, flavones, and isoflavones), hydroxycinnamic acids, hydroxybenzoic acids, and phenols at 10–30 times the rates observed in other oilseeds [72].

### 4.1. Metabolites Related to Abiotic Resistance

#### 4.1.1. Macronutrient Stress Response

To assess whether transcriptomic and metabolomic profiling of *B. napus* roots can characterize the effect of individual deficiency of N, P, K, S, Mg, and Ca, Courbet et al. [73] demonstrated that, prior to any visible phenotypic response, all macronutrient deprivations induced substantial transcriptomic and metabolomic modulation across diverse metabolic pathways, with certain alterations being consistently observed across every treatment condition. Furthermore, using a metabolic approach, the authors identified a panel of 38 biochemicals discriminating among different macronutrient deprivations, which may serve as potential molecular indicators for diagnosing macronutrient deficiency [73].

In another study, Peng et al. [74] systematically characterized the lipidome and metabolome responses of rapeseed to N starvation. They found that N starvation severely stunted plant growth and significantly reduced the levels of the majority of amino acids. The level of monogalactosyldiacylglycerol (MGDG) significantly declined in both leaves and roots, whereas digalactosyldiacylglycerol (DGDG) levels significantly decreased only in roots, leading to a marked reduction in the MGDG/DGDG ratio under N starvation. Concurrently, the abundances of phosphatidylglycerol, sulfoquinovosyl diacylglycerol, and glucuronosyl diacylglycerol were diminished to variable degrees. Furthermore, N deficiency altered the levels of metabolites associated with the Calvin cycle, TCA (tricarboxylic acid) cycle, and energy metabolism. Those findings indicate that N deprivation induces perturbations in both the metabolism of membrane lipids and carbon metabolic pathways [74].

Similarly, when utilizing a multi-omics approach to elucidate how *B. rapa* adapts to N-deficient conditions, Gong et al. [75] uncovered 1917 and 1481 DEGs (differentially expressed genes) in leaves and roots, respectively. These DEGs were mainly linked to photosynthesis, oxidative stress responses, and amino acid metabolism, and corresponded with significant metabolic changes in phenylpropanoid biosynthesis, amino acid metabolism, and energy pathways. By integrating metabolomic and transcriptomic data using WGCNA, core gene–metabolite modules implicated in the adaptive response to N stress were identified, offering valuable insights that may serve to enhance N use efficiency [75].

On the contrary, excessive NH_4_^+^ uptake is toxic to plants, leading to leaf chlorosis, abnormal root structure, and stunted growth, amongst other negative consequences [76]. Comprehensive metabolomic analysis corroborated that Gln metabolism plays a significant role in mediating NH_4_^+^ tolerance. Furthermore, the N-glycosylation pathway, particularly mannose metabolism, was significantly induced by NH_4_^+^; mannose concentrations were 2.75 times higher in T5 shoots relative to S211 shoots under NH_4_^+^ treatment. This finding suggests that mannose may not only serve as a metabolome marker but also play a role in the physiological adaptive responses that confer NH_4_^+^ tolerance in rapeseed [77].

#### 4.1.2. Cold Temperature Response

Cold temperatures in winter hinder seed germination and the early growth of rapeseeds, ultimately resulting in reduced yields. When characterizing cold stress responses using five winter and five spring ecotypes of *B. napus*, Jian et al. [49] found that lipids, glycerophospholipids, abscisic acid (ABA), and secondary metabolism may have distinct functions in winter and spring ecotypes under cold stress. Similarly, arachidonic acid and proline were reported to be up-accumulated in the cold-tolerant *B. napus* variety subjected to cold stress [78], while indole acetic acid (IAA) was involved in low-temperature germination, conferring an increased germination rate under cold conditions [79]. Moreover, metabolome profiling indicated that phenylalanine, amino sugar, nucleotide sugar, purine, and flavonoid biosynthesis were the top-enriched pathways in rapeseed seedlings after cold acclimation (CA). Concurrent analysis of transcriptomic and metabolomic changes revealed the dynamic nature of specific gene–metabolite interactions in rapeseed. Correlation analysis between DAMs and DEGs showed that *RAP*, *DREB*, *ZAT12*, *TCP2*, *ASP1*, *ASP4*, and *ASP3*, along with 2-glycitein, N-acetyl-D-glucosamine, and 3-hydroxybenzyl alcohol glucoside, played important roles in rapeseed post CA. Collectively, these findings elucidate the molecular regulation mechanisms underpinning the capacity of rapeseed to tolerate subzero temperatures after cold acclimation [80].

On the basis of transcriptomic and metabolomic changes in the freezing-treated Tibetan turnip, Yin et al. [81] found that *BrrADC2.2*, the arginine decarboxylase gene, showed an accumulation pattern that paralleled putrescine levels. Furthermore, the inducer of CBF Expression 1 (*Brr*ICE1.1) was demonstrated to bind directly to the promoter of *BrrADC2.2*, thereby activating its expression and subsequently enhancing putrescine accumulation. The contribution of putrescine to the freezing tolerance in turnips was further examined through the exogenous treatment of putrescine alongside its specific inhibitor, DL-α-(difluoromethyl) arginine (DFMA). Additionally, using transcriptomics and targeted metabolomics analyses, the authors demonstrated that *Brr*ICE1.1 binds to the promoters of both *BrrADC2.2* and *CBFs* to participate in the freezing tolerance in turnips [81].

#### 4.1.3. Water Stress Response

Water is essential for plant growth and development. However, when soil is extremely dry or exceeds its maximum water-holding capacity, plants suffer from drought or waterlogging stress, respectively, severely impairing normal growth. Under continuous rainfall and waterlogging stress, specific metabolites associated with flavonoid biosynthesis and vitamin B6 homeostasis, including epiafzelechin and naringenin, were significantly enhanced in G230 leaves. Conversely, a significant down-regulation of pyridoxine phosphate was detected exclusively in the root and leaf tissues of G218 [82]. Furthermore, foliar application of vitamin B6 was shown to transiently but effectively improve waterlogging tolerance in G218. Collectively, these findings show that the flavonoid biosynthesis and vitamin B6 metabolic pathways are essential for tolerance to waterlogging and resistance to hypoxia stress in *B. napus* [82].

The principal component analysis (PCA) revealed that epicatechin and chlorogenic acid under well-watered conditions, and myricetin under drought stress, were the key components contributing to radical scavenging activity (RSA). Hierarchical cluster analysis (HCA) revealed that vanillic acid, gallic acid, catechin, and myricetin were closely associated under both irrigation regimes. Of the 60 candidate genes (CGs) identified by GWAS, 18 were involved in stress-induced pathways, flavonoid modifications, and the phenylpropanoid pathway [83]. Among these CGs, *PAL1*, *UGT89B1*, *CHI*, *CCR1*, *FLS3*, and *CYP75B137* participated in flavonoid biosynthesis. RNA-seq showed that transcription of early flavonoid biosynthesis-related genes (*PAL*, *CHI*, and *CYP75B137*), as well as genes involved in later stages (*FLS3*, *CCR1*, and *UGT89B1*), were increased under drought stress. Furthermore, *ERF119* and *NAC035*, which are associated with flavonoid and phenolic acid regulation, were also upregulated under drought stress [83].

To develop a microbial biocontrol agent against drought stress, Kim et al. [84] identified lactic acid as a metabolite indicator that enhanced drought tolerance in *B. rapa* when treated with *Lysinibacillus capsici* TT41. Moreover, exogenous application of lactic acid alone was sufficient to effectively induce drought stress tolerance. Furthermore, the exogenous application of fullerol to the foliage of *B. napus* seedlings subjected to drought stress resulted in the predominant enrichment of both drought- and fullerol-responsive differentially expressed genes and differentially accumulated metabolites within the Kyoto Encyclopedia of Genes and Genomes (KEGG) pathways, pertaining to amino acid metabolism (notably amino acid biosynthesis and the metabolism of arginine and proline), carbohydrate metabolism (including carbon metabolism and galactose metabolism), and secondary metabolite biosynthesis [85]. Regarding carbohydrate metabolism, drought decreased the accumulation of oligosaccharides (e.g., sucrose) but increased that of monosaccharides (e.g., mannose and myo-inositol). In terms of amino acid metabolism, drought stress reduced the accumulation of amino acids, such as Phe and Trp, while increasing that of Glu and Pro [85]. For secondary metabolite metabolism, *B. napus* under soil drying showed reductions in flavonoids and phenolics, such as trans-3-coumaric acid and hyperoside. However, in fullerol-treated *B. napus* under drought conditions, carbohydrate accumulation remained largely unchanged. Under water shortage, fullerol treatment decreased the accumulation of certain amino acids (e.g., Pro) but enhanced that of flavonoids and phenolics, such as trans-3-coumaric acid and luteolin. These findings indicate that fullerol alleviates the inhibitory impact of drought stress on the levels of flavonoids and phenolics, thereby enhancing drought tolerance in *B. napus* [85].

#### 4.1.4. Salt Stress Response

Soil salinization is a major environmental issue that constrains crop growth and productivity. To elucidate the tolerance mechanisms of rapeseed under alkaline salt stress, Wang et al. [51] conducted a metabolomic analysis, and found increased accumulation of an array of amino acids and fatty acids, including Pro, Glu, His, Phe, citrulline, Tyr, Trp, linoleic acid and derivative, linolenic acid, oleic acid, erucic acid, and neuronic acid, whereas the metabolism of carbohydrates (such as sucrose, trehalose, UDP-D-galactose, mannose, fructose, and glucose 6-phosphate) was reduced, indicating the important roles of fatty acid and amino acids metabolism under alkaline salt stress.

Through functional enrichment analysis of DEGs, DAMs, and differentially expressed proteins (DEPs), key factors governing the response of *B. napus* to salt stress were identified [86]. The results showed that ABA and Jasmonic acid (JA) are the two key hormones responding to salt stress. Salt stress for 24 h represents an important milestone, as *B. napus* adjusts multiple pathways at this time point to avoid a hyper-responsive reaction to salt stress and unnecessary energy consumption. The elevated expression of *BnPP2C* provides tangible evidence of this regulation. Upon exposure to salt stress, JA and ABA act synergistically to reduce salt-induced damage in *B. napus*. The upregulated expression of all *BnaJAZs* following salt stress highlights the indispensable role of JA in salt stress responses. Additionally, several metabolites—such as N-acetyl-5-hydroxytryptamine, L-Cys, and L-(+)-Arg—play critical roles in maintaining reactive oxygen species (ROS) homeostasis. Proteins including catalase-3, cysteine desulfurase, HSP90, and P450_97A3 were identified as the most critical differential proteins involved in the response to salt stress [86].

#### 4.1.5. Al and Cd Toxicity Response

Aluminum (Al) toxicity is a significant abiotic stress factor in acid soil areas, which impairs root absorption of water and nutrients, thereby retarding crop growth. Through the integration of metabolomics with QTL mapping and transcriptomics of 138 recombinant inbred lines, 138 hub genes were identified as involved in the root response of rapeseed to aluminum stress, with primary functions in the metabolism of lipids, carbohydrates, and secondary metabolites [50].

Meanwhile, for the phytoremediation of cadmium (Cd)-contaminated soils, *B. napus* has great potential due to its large biomass production and strong metal accumulation capacity. Using fine isolation of cell components combined with ionomics, Zhang et al. [87] revealed that more Cd accumulated in the shoot vacuoles and root pectins of a resistant genotype than in a sensitive one. Transcriptomics-assisted gene co-expression networks identified *BnaCn.ABCC3* and *BnaA8.PME3* as the central members involved in determining Cd resistance (PCR) in rapeseed. High-resolution metabolic profiles further demonstrated a greater accumulation of shoot Cd chelates, as well as stronger biosynthesis and higher demethylation of root pectins, in the resistant genotype compared with the sensitive one.

In addition, combining metabolomic profiling with transient overexpression experiments, Liu et al. [88] demonstrated that *BnaBAHD120* and *BnaBAHD040*, both of which are upregulated during late seed development, greatly boosted acylated metabolite accumulation, thereby improving biotic and abiotic stress resistance. In a separate study, Wei et al. [89] performed transient overexpression of *BnaBGLU77* in *Nicotiana benthamiana* coupled with untargeted metabolomic profiling, revealing pronounced modulatory influences on the turnover (both degradation and accumulation) of β-glucosidic compounds [88]. These findings suggest possible functions of the *BnaBGLU77*-encoded protein in metabolic homeostasis and stress responses.

### 4.2. Metabolites Related to Biotic Resistance

#### 4.2.1. *Sclerotinia sclerotiorum* Infection

The hemibiotrophic nature of *S. sclerotiorum* and its long persistence in soil as sclerotia make this pathogen extremely difficult to manage through conventional agronomic practices. Therefore, developing alternative strategies requires a fundamental understanding of the pathogen and the host’s pathogenesis processes. Using proteomic and metabolomic analyses, Teng et al. [90] showed that thiamine synthesis was activated in *B. rapa* during *S. sclerotiorum* infection and contributed to the host defense. Integrated proteomic and metabolomic analysis further revealed that melatonin (MT) treatment activated amino acid metabolism. Following MT treatment in infected *B. rapa* leaves, ATP content and the activity of antioxidant enzymes, cysteine synthase, sulfur transfer-related proteins, and glucosinolates (GSLs) were all increased. Taken together, these results indicate that *B. rapa* leaves promote the formation of thiamine, which contributes to the plant’s defense against infection by *S. sclerotiorum*. Moreover, MT further drives ATP-dependent antioxidant activation and GSL biosynthesis in *B. rapa*, thereby effectively inhibiting *S. sclerotiorum* infection [90].

Furthermore, Qasim et al. [91] found that in resistant *B. napus* lines, genes involved in secondary metabolite biosynthetic pathways—including those for phenylpropanoids, glucosinolates, flavonoids, Arg, Ala, Asp, and Glu—were relatively more induced in response to *S. sclerotiorum* infection compared with susceptible lines. Barreda et al. [92] observed that long-chain (C8–C11) methylsulfinylalkyl glucosinolates (GSLs) mainly accumulated in the seed coat and endosperm, whereas mid-/short-chain (C3–C7) methylsulfinylalkyl GSLs accumulated in the embryo. Elucidating the spatial dynamics of specialized metabolites within seeds yields valuable insights that can inform and guide the rational development of crop varieties with optimized partitioning of beneficial versus potentially toxic metabolites, thereby improving seed nutritional profiles [92]. For more details on the *Brassica*–*S. sclerotiorum* pathosystem deciphered using the multi-omics strategies, please refer to the review paper by Singh et al. [25].

#### 4.2.2. *Leptosphaeria maculans* Infection

Blackleg, caused by *L. maculans*, is a major disease of *B. napus* commonly found in Europe, Canada, and Australia. When metabolic profiles were analyzed for *L*. *maculans*, infected resistant (R) and susceptible (S) *B. napus* cultivars at 21 °C and 28 °C, Amjadi et al. [55] identified that pathways involved in lipid metabolism, amino acid metabolism, alkaloid biosynthesis, flavonoid biosynthesis, and phenylpropanoid biosynthesis were associated with combined heat and pathogen stresses. In addition, carotenoid biosynthesis, lysine biosynthesis, and pyrimidine metabolism were classified as heat-stress-specific mechanisms that may contribute to the breakdown of pathogen resistance.

#### 4.2.3. *Xanthomonas campestris* Infection

When investigating *X. campestris* pv. *campestris* (*Xcc*)-induced transcriptomic and metabolic changes in the leaves of two rapeseed varieties, Westar (susceptible) and ZS5 (resistant), Zhou et al. [93] showed that *Xcc* infection triggered greater transcriptomic and metabolic changes in Westar than in ZS5. Transcriptomics revealed enrichment of cutin/suberine/wax biosynthesis, phenylpropanoid biosynthesis, Phe metabolism, and Trp metabolism pathways in both varieties. Indolic glucosinolates and IAA are two metabolites derived from Trp. Following infection, indolic glucosinolate pathway gene expression and glucosinolate levels were both significantly elevated in the two varieties. In addition, exogenous IAA promoted black rot, whereas an IAA synthesis inhibitor attenuated it in both susceptible and resistant rapeseed varieties [93].

#### 4.2.4. *Agrobacterial tumefaciens* Infection

When using ^1^H nuclear magnetic resonance (NMR) spectroscopy combined with multivariate data analysis, Simoh et al. [94] found a clear differentiation in the metabolite profiles of *B. rapa* leaves infected with the disarmed strain LBA4404 versus those infected with tumor-inducing nopaline (a conjugate of α-ketoglutarate and Arg) and octopine (a conjugate of Arg and pyruvate) strains. The observed differences were particularly evident in the contents of flavonoids, phenylpropanoids, sugars, and free amino/organic acids. However, partial least squares discriminant analysis (PLS-DA) of each infection type suggested that, overall, some phenylpropanoids and flavonoids were suppressed following infection, indicating that LBA4404 and tumor-inducing strains differentially affect *B. rapa* metabolite profiles [94].

#### 4.2.5. *Plasmodiophora brassicae* Infection

Clubroot disease, caused by the obligate biotrophic pathogen *P. brassicae*, affects all *Brassicaceae* species. In a study investigating the dynamics and diversity of metabolic responses to infection, Wagner et al. [95] reported that early secondary infection-induced accumulation of amino acids, glutathione, and S-methylcysteine correlates with clubroot susceptibility in *B. napus*. Furthermore, a common metabolic hallmark of clubroot susceptibility is the accumulation of pathogen-synthesized trehalose, which occurs mostly during the later stages of infection [96,97].

To tackle the complexity inherent in metabolic responses of *B. napus* roots following infection by *P. brassicae*, Wagner et al. [98] conducted metabolome profiling and pathogen quantification in a segregating progeny to map QTLs involved in resistance and metabolic adjustments. The authors identified a possible role for gluconasturtiin and two unknown metabolites in the resistance conferred by two QTLs located on chromosomes C03 and C09, respectively. Only glycine and glutathione were simultaneously linked to the three major resistance QTLs, suggesting they play a central role in host–pathogen interactions. By contrast, several genotype-specific infection responses were found to be genetically independent of resistance or susceptibility. To summarize, some resistance-related metabolites that responded to abiotic/biotic stresses, which were identified based on metabolomics in rapeseeds, are listed in Table 2.

## 5. Conclusions and Future Perspective

To date, multi-omics analysis integrating transcriptomics, metabolomics, proteomics, and genomics has become an effective tool for screening and analyzing key genes involved in various metabolic pathways in plants [20,101,102]. Furthermore, gene-to-metabolite networks are now well understood, particularly in the flavonoid biosynthesis pathways of many species, including *A. thaliana* [103], red and green kale (*Brassica oleracea*) [104], red mustard greens [105], and purple tumorous stem mustard [20].

In rapeseed, the widespread application of metabolomics and its recent integration with transcriptomics, proteomics, and genomics have enabled the identification of an increasing number of important agronomic, physiological, and nutritional traits, as well as genetic associations between markers and metabolites. These include embryo development, seed oil content, seed coat color, health-related phytochemicals, and metabolites associated with biotic and abiotic stress resistance in rapeseed, opening new horizons for future research. Omics technologies further facilitate a deeper understanding of the host, the pathogen, and the molecular interplay between these two species, which provides a promising strategy for the development of high-quality *Brassica* crops that exhibit broad-spectrum resilience to diverse pathogens, while maintaining both yield and crop quality [88,106].

However, many challenges and constraints still exist in the study of rapeseed metabolites using metabolomics approaches. First, it is crucial to eliminate the influence of external factors, such as growth environment and experimental methods, on the plant metabolome to ensure systematic consistency and accurate results. Second, although the rapid development of various metabolomics technologies has enabled the analysis of a vast number of compounds, the data generated by metabolomics research can only cover a portion of the plant metabolome due to the extreme complexity of metabolites in rapeseed and the incompleteness of relevant databases. Third, for identified metabolites, their biological significance within the plant body is extremely complex, and current approaches still cannot provide a comprehensive understanding of the in planta mechanisms underlying growth, development, and stress resistance.

Nevertheless, with the integration of multi-omics and systems biology data, including metabolite genome-wide association studies, metabolite transcriptome-wide association studies, and other approaches, a more comprehensive biological network of plant metabolites can be constructed. This enables a deeper understanding of the functions and mechanisms of metabolites in planta. In the future, with the advances in new marker discovery, special trait prediction, metabolic pathway selection, and even the application of artificial intelligence, metabolomics, when integrated with other omics, will boost its great potential for breeding in rapeseeds. Ultimately, we can breed high-quality, resistant *Brassica* crops in a manner that is more comprehensive, precisely targeted, and accurate through marker-assisted selection and molecular design breeding, leading to the development of stable, high-yield, and high-quality new rapeseed varieties.

## Figures and Tables

**Table 1:** Typical metabolite types unveiled currently by metabolomics in rapeseeds.

Name	Number of Types	Analytical Methods	Rapeseed Types	References
Nucleic acid derivatives	~52	LC–MS, GC-MS, UPLC-MS/MS, UPLC-Q-TOF/MS	*B. rapa*, *B. napus*	[28,31,42,48,49,50]
Amino acid derivatives	~81	LC–MS, GC-MS, UPLC-MS/MS, UPLC-Q-TOF/MS, LC-QTOF-MS	*B. rapa*, *B. napus*	[28,31,42,46,48,49,50,51]
Fatty acids	~57	GC-MS, UPLC-Q-TOF/MS, UHPLC −MS/MS, UHPLC−HESI−MS/MS	*B. rapa*, *B. napus*	[31,41,48,51,52]
Lipids	~96	LC–MS, LC-MS/MS, UPLC-MS/MS, UPLC-Q-TOF/MS, UPLC-HESI-MS/MS	*B. rapa*, *B. napus*, *B. juncea*	[28,30,31,45,46,48,49,50,53,54,55]
Organic acids	~74	GC-MS, UPLC-MS/MS, UPLC-Q-TOF/MS, UPLC-HESI-MS/MS	*B. rapa*, *B. napus*, *B. juncea*	[31,42,45,48,49]
Alkaloids	~45	UPLC-MS/MS, UPLC-Q-TOF/MS, UHPLC−HESI−MS/MS	*B. rapa*, *B. napus*	[31,48,50,52]
Phenylethanoids and Phenylpropanoids	~55	LC–MS, UPLC-Q-TOF/MS	*B. napus*	[28,48]
Terpenoids	~42	LC–MS, UPLC-MS/MS, UPLC-Q-TOF/MS, UHPLC−HESI−MS/MS	*B. rapa*, *B. napus*	[28,31,48,52]
Flavonoids	~124	LC–MS, UPLC-MS/MS, LC-QTOF-MS, UHPLC-MS/MS, UPLC-QTOF-MS/MS, UPLC-HESI-MS/MS	*B. rapa*, *B. napus*, *B. juncea*	[28,29,30,31,33,45,46,47,48,53,56]
Phenolic acids	~82	UPLC-MS/MS, UPLC-QTOF-MS/MS, UPLC-HESI-MS/MS	*B. rapa*, *B. napus B. juncea*	[29,30,31,45,56]
Lignans and coumarins	~33	UPLC-MS/MS, UPLC-Q-TOF/MS, UPLC-QTOF-MS/MS	*B. rapa*, *B. napus*, *B. juncea*	[29,31,50]
Tannins	~3	UPLC-MS/MS, UPLC-QTOF-MS/MS	*B. rapa*, *B. napus*, *B. juncea*	[29,31]
Glucosinolates	~33	LC–MS, UPLC-MS/MS, UPLC-HESI-MS/MS	*B. rapa*, *B. juncea*	[30,31,33,45,57]

**Table 2:** Some resistance-related metabolites identified based on metabolomics in rapeseeds.

Metabolites	Stress Types	Stress Response	Rapeseed Type	References
Mannose	Abiotic	NH_4_^+^ tolerance, drought, alkaline salt stress	*B. rapa*, *B. napus*	[51,77,85]
Trehalose	Abiotic, Biotic	Alkaline salt stress, cold stress, *P. brassicae*	*B. napus*	[98,99]
Citric acid	Biotic	*P. brassicae*	*B. napus*	[98]
Lactic acid	Abiotic	Drought	*B. rapa*	[84]
Oxaloacetate	Abiotic	N starvation	*B. rapa*	[75]
Caffeic acid	Abiotic	N starvation	*B. rapa*	[75]
Linoleic acid	Abiotic	Alkaline salt stress	*B. napus*	[51,69]
Linolenic acid	Abiotic	Alkaline salt stress	*B. napus*	[51]
Erucic acid	Abiotic	Alkaline salt stress	*B. napus*	[51]
Oleic acid	Abiotic	Alkaline salt stress	*B. napus*	[51]
Arachidonic acid	Abiotic	Cold stress	*B. napus*	[78]
Phospholipids	Abiotic	N, Mg, Ca deprivation, cold stress	*B. napus*	[49,73]
MGDG/DGDG	Abiotic	N starvation	*B. napus*	[74]
Glutamine/glutamate	Abiotic, biotic	N starvation, NH_4_^+^ tolerance, drought, alkaline salt stress, *S. sclerotiorum*	*B. napus*, *B. rapa*	[51,73,77,85,91]
Tyr	Abiotic	N starvation, alkaline salt stress	*B. rapa*, *B. napus*	[51,75]
Ala	Biotic	*S. sclerotiorum*, *P. brassicae*	*B. napus*	[91,98]
Gly	Biotic	*P. brassicae*	*B. napus*	[98]
Thr	Biotic	*P. brassicae*	*B. napus*	[98]
Ile	Biotic	*P. brassicae*	*B. napus*	[98]
Val	Biotic	*P. brassicae*	*B. napus*	[98]
Phe	Abiotic, biotic	Drought, alkaline salt stress, cold stress, *P. brassicae*	*B. napus*	[51,85,98,99]
Trp	Abiotic, biotic	Drought, alkaline salt stress; *P. brassicae*	*B. napus*	[51,77,85,98]
Pro	Abiotic	Drought, cold, salt, alkaline salt stress	*B. napus*	[51,78,85,100]
His	Abiotic	Alkaline salt stress	*B. napus*	[51]
Ser	Abiotic	Cadmium tolerance	*B. napus*	[87]
Met	Abiotic	Cadmium tolerance	*B. napus*	[87]
Cys	Abiotic	Salt Stress, cadmium tolerance	*B. napus*	[86,87,95]
S-methylcysteine	Biotic	*P. brassicae*	*B. napus*	[95,98]
Arg	Abiotic, biotic	Salt Stress, *S. sclerotiorum*	*B. napus*	[86,91]
Citrulline	Abiotic	Alkaline salt stress	*B. napus*	[51]
Gluconasturtiin	Biotic	*P. brassicae*	*B. napus*	[98]
Putrescine	Abiotic	Freezing tolerance	*B. rapa*	[81]
Glutathione	Abiotic, biotic	Cadmium tolerance, *P. brassicae*	*B. napus*	[87,95,98]
N-acetyl-5-hydroxytryptamine	Abiotic	Salt Stress	*B. napus*	[86]
N-acetyl-D-glucosamine	Abiotic	Cold acclimation	*B. rapa*	[80]
Phenylpropanoids	Abiotic, biotic	N, P, K, S, Mg, Ca deprivation, *S. sclerotiorum*, *A. tumefaciens*	*B. napus*, *B. rapa*	[73,75,92,94]
2-glycitein	Abiotic	Cold acclimation	*B. rapa*	[80]
3-hydroxybenzyl alcohol glucoside	Abiotic	Cold acclimation	*B. rapa*	[80]
Myo-inositol	Abiotic	Drought	*B. napus*	[85]
Vanillic acid	Abiotic	Drought	*B. napus*	[83]
Myricetin	Abiotic	Drought	*B. napus*	[83]
Gallic acid	Abiotic	Drought	*B. napus*	[83]
Catechin	Abiotic	Drought	*B. napus*	[83]
Hyperoside	Abiotic	Drought	*B. napus*	[85]
trans-3-coumaric acid	Abiotic	Drought	*B. napus*	[85]
Luteolin	Abiotic	Drought	*B. napus*	[85]
Naringenin	Abiotic	Waterlogging	*B. napus*	[82]
Epiafzelechin	Abiotic	Waterlogging	*B. napus*	[82]
Pyridoxine phosphate	Abiotic	Waterlogging	*B. napus*	[82]
ABA	Abiotic	Salt stress, cold stress	*B. napus*	[49,86]
JA	Abiotic	Salt stress	*B. napus*	[86]
IAA	Abiotic, biotic	Cold stress, *X. campestris*	*B. napus*	[79,93]
Nicotinamide	Abiotic	Cadmium tolerance	*B. napus*	[87]
Glucosinolates	Biotic	*S. sclerotiorum*, *X. campestris*, *P. brassicae*	*B. rapa*, *B. napus*	[90,91,93,98]
Thiamine	Biotic	*S. sclerotiorum*	*B. rapa*	[90]

Note: MGDG: monogalactosyldiacyglycerol, and DGDG: digalactosyldiacylglycerol.

## Data Availability

The original contributions presented in this study are included in the article. Further inquiries can be directed to the corresponding author.
